# On the necessity to include arterial pre-stress in patient-specific simulations of minimally invasive procedures

**DOI:** 10.1007/s10237-023-01789-0

**Published:** 2023-12-08

**Authors:** Anna Ramella, Vittorio Lissoni, Sara Bridio, Jose Felix Rodriguez Matas, Santi Trimarchi, Benedetta Grossi, Giulio G. Stefanini, Francesco Migliavacca, Giulia Luraghi

**Affiliations:** 1 Computational Biomechanics Laboratory – LaBS, Department of Chemistry, Materials and Chemical Engineering ‘Giulio Natta’, Politecnico di Milano, Piazza L. da Vinci 32, 20133 Milan, Italy; 2 Section of Vascular Surgery, Cardio Thoracic Vascular Department, Foundation IRCCS Ca’ Granda Ospedale Maggiore Policlinico, Via Francesco Sforza 35, Milan, Italy; 3 Department of Clinical Sciences and Community Health, University of Milan, Via Della Commenda 19, 20122 Milan, Italy; 4 Department of Biomedical Sciences, Humanitas University, Via Rita Levi Montalcini 4, 20072 Pieve Emanuele, Milan, Italy; 5 IRCCS Humanitas Research Hospital, Via Alessandro Manzoni 56, 20089 Rozzano, Milan, Italy

**Keywords:** Transcatheter aortic valve implantation, Thoracic endovascular aortic repair, Finite element analysis, Aortic valve, Stent-graft

## Abstract

Transcatheter aortic valve implantation (TAVI) and thoracic endovascular aortic repair (TEVAR) are minimally invasive procedures for treating aortic valves and diseases. Finite element simulations have proven to be valuable tools in predicting device-related complications. In the literature, the inclusion of aortic pre-stress has not been widely investigated. It plays a crucial role in determining the biomechanical response of the vessel and the device–tissue interaction. This study aims at demonstrating how and when to include the aortic pre-stress in patient-specific TAVI and TEVAR simulations. A percutaneous aortic valve and a stent-graft were implanted in aortic models reconstructed from patient-specific CT scans. Two scenarios for each patient were compared, i.e., including and neglecting the wall pre-stress. The neglection of pre-stress underestimates the contact pressure of 48% and 55%, the aorta stresses of 162% and 157%, the aorta strains of 77% and 21% for TAVI and TEVAR models, respectively. The stent stresses are higher than 48% with the pre-stressed aorta in TAVI simulations; while, similar results are obtained in TEVAR cases. The distance between the device and the aorta is similar with and without pre-stress. The inclusion of the aortic wall pre-stress has the capability to give a better representation of the biomechanical behavior of the arterial tissues and the implanted device. It is suggested to include this effect in patient-specific simulations replicating the procedures.

**Supplementary Information:**

The online version contains supplementary material available at 10.1007/s10237-023-01789-0.

## Introduction

Patients’ physiological and pathological cardiovascular conditions have been investigated in recent years by replicating in silico clinical procedures. Computational models, indeed, when verified and validated, provide a reliable and non-invasive tool to obtain data that would otherwise be difficult to get through in vivo or in vitro measurements. Help in the development of new medical devices, assistance in surgical planning, and exploration of tissue growth and remodeling resulting from pathological conditions (Torii et al. 2007; Hsu and Bazilevs 2011; Peirlinck et al. 2018) are among the goals of in silico medicine. Among the numerous in silico cardiovascular studies replicating a clinical scenario, one of the most common simplifications is using patient-specific arterial models without accounting for the initial stress state of the vessel wall (Perrin et al. 2015a, b; Lin et al. 2017; Mao et al. 2018; Derycke et al. 2019; Romarowski et al. 2019; Bianchi et al. 2019; Luraghi et al. 2019, 2020; Basri et al. 2020; Bosi et al. 2020). Typically, patient-specific anatomies are reconstructed using segmentation techniques from clinical images like computed tomography angiography (CTA) or magnetic resonance (MR) acquired in the diastolic phase. Hence, the reconstructed geometry corresponds to a pressurized (stressed) configuration. Nevertheless, since diagnostic images lack information on the stress state of the vessel walls, additional pre-processing techniques are required to incorporate this aspect into the models.

Neglecting wall pre-stress is particularly critical when dealing with clinical procedures such as Transcatheter Aortic Valve Implantation (TAVI) and Thoracic Endovascular Aortic Repair (TEVAR). Its omission can result in inaccurate prediction of vessels’ mechanical behavior, vessel–device interaction, and device performance once deployed in the anatomies. TAVI is applied to treat severe valvular diseases (e.g., aortic stenosis). It is based on implanting a self-expandable stented valve through the subclavian, carotid, or femoral artery using a catheter (Leon et al. 2010). TEVAR is used to treat thoracic aortic pathologies (e.g., aneurysms, ulcerations, dissections) and consists in placing a self-expandable stent-graft into the diseased region to restore the lumen. The device is delivered to the aorta thanks to a catheter introduced through the femoral artery (Findeiss and Cody 2011).

Several studies in the literature replicated the two mentioned endovascular procedures in patient-specific anatomies with finite element (FE) simulations to investigate the outcomes and risks of the procedures (about 20 papers in the last 2 years). However, only a few TEVAR studies consider aortic wall pre-stress (Desyatova et al. 2020; Concannon et al. 2021; Kan et al. 2021; Shahbazian et al. 2022), while, to the best of our knowledge, studies accounting for aortic root pre-stress in TAVI procedure simulations are missing. Concannon et al. (2021) conducted a study comparing the outcomes of TEVAR procedures in patients of different ages. The unloaded configuration at 0 mmHg of the aorta was determined by solving an inverse problem that mapped the segmented deformed configuration back to the undeformed one by knowing the diastolic pressure. Then, they included the wall pre-stress by loading the undeformed vessel to the diastolic condition. TEVAR simulations accounting for pre-stress were also described in the works by Kan et al. (2021) on patient-specific aortic dissection models. In Kan et al., aortic pre-stress was iteratively computed by loading the vessel segmented from CTA images to diastolic condition and using the resulting wall stress as the initial wall stress state for the following iteration. This iterative process ended when the nodal displacement resulting from the pressurization was lower than the CTA in-plane resolution, meaning that the wall stress state was in equilibrium with the external load. Shahbazian et al. (2022) incorporated the pre-stress in idealized thoracic aortic models by pressurizing the segmented aortic geometry up to the diastolic pressure, then subtracted the resulting nodal displacements incrementally from the initial geometry until the residual difference between the current and initial configurations was minimized. Pre-stretch in addition to pre-stress was only considered by Desyatova et al. (2020). They studied the effect of longitudinal stretch on aorta mechanics before and after TEVAR interventions. The aorta was modeled as a nonlinear anisotropic material, and the pre-stretch and pre-stress, considered age dependent, were calculated by deforming the vessel with the application of uniform internal pressure and constraining the brachiocephalic arteries with spring-like elements. They found that incorporating the longitudinal pre-stretch increases intramural stress after TVAR for young individuals, whereas it has no effect on elder patients.

None of the aforementioned studies carefully explores and quantifies the influence of vessel pre-stress in TAVI and TEVAR modeling. Therefore, this paper aims at demonstrating the impact of including aortic wall pre-stress in TAVI and TEVAR structural FE simulations, providing a precise quantification of mechanical markers related to post-implantation surgical outcomes, device–tissue interaction, and potential complications. The endovascular interventions were simulated for patient-specific anatomies where pre-stress is calculated with an inverse elastostatic method, both for the TAVI and TEVAR scenarios. We demonstrate important changes in the device performance and vessel stresses and strains post-implantation if pre-stress is considered.

## Methods

### Patient data

The surgical procedures were simulated in two TAVI and two TEVAR patients, whose data were obtained retrospectively.

TAVI Patient 1 (TAVI-pat1) was an 87-year-old man who presented a symptomatic aortic calcified stenosis with a mean aortic transvalvular gradient of 43 mmHg. TAVI Patient 2 (TAVI-pat2) was a 67-year-old man with symptomatic aortic calcified stenosis with a mean aortic transvalvular gradient of 42 mmHg. In both cases, TAVI was performed at Humanitas Research Hospital, Milan, Italy with CoreValve Evolut R® size 29 (Medtronic, Inc., MN, U.S.A.). TEVAR Patient 1 (TEVAR-pat1) was a 63-year-old man and presented a Penetrating Aortic Ulceration (PAU) in his left hemi-aortic arch (diameter of 26 by 32 mm in axial and sagittal sections). The patient has a bovine aortic arch with a common origin of the brachiocephalic trunk and left common carotid artery. TEVAR Patient 2 (TEVAR-pat2) was an 81-year-old man with a PAU in the distal arch (diameters of 31 by 34 mm in axial and sagittal sections). In both cases, TEVAR was performed at the Fondazione IRCCS Ca’ Granda Ospedale Maggiore Policlinico, Milan, Italy with a 34 × 34 × 100 proximal free-flow Valiant Captivia® stent-grafts (Medtronic, Inc., MN, U.S.A.). Approval for this specific study was waived by the local ethical committees.

**Fig. 1:**
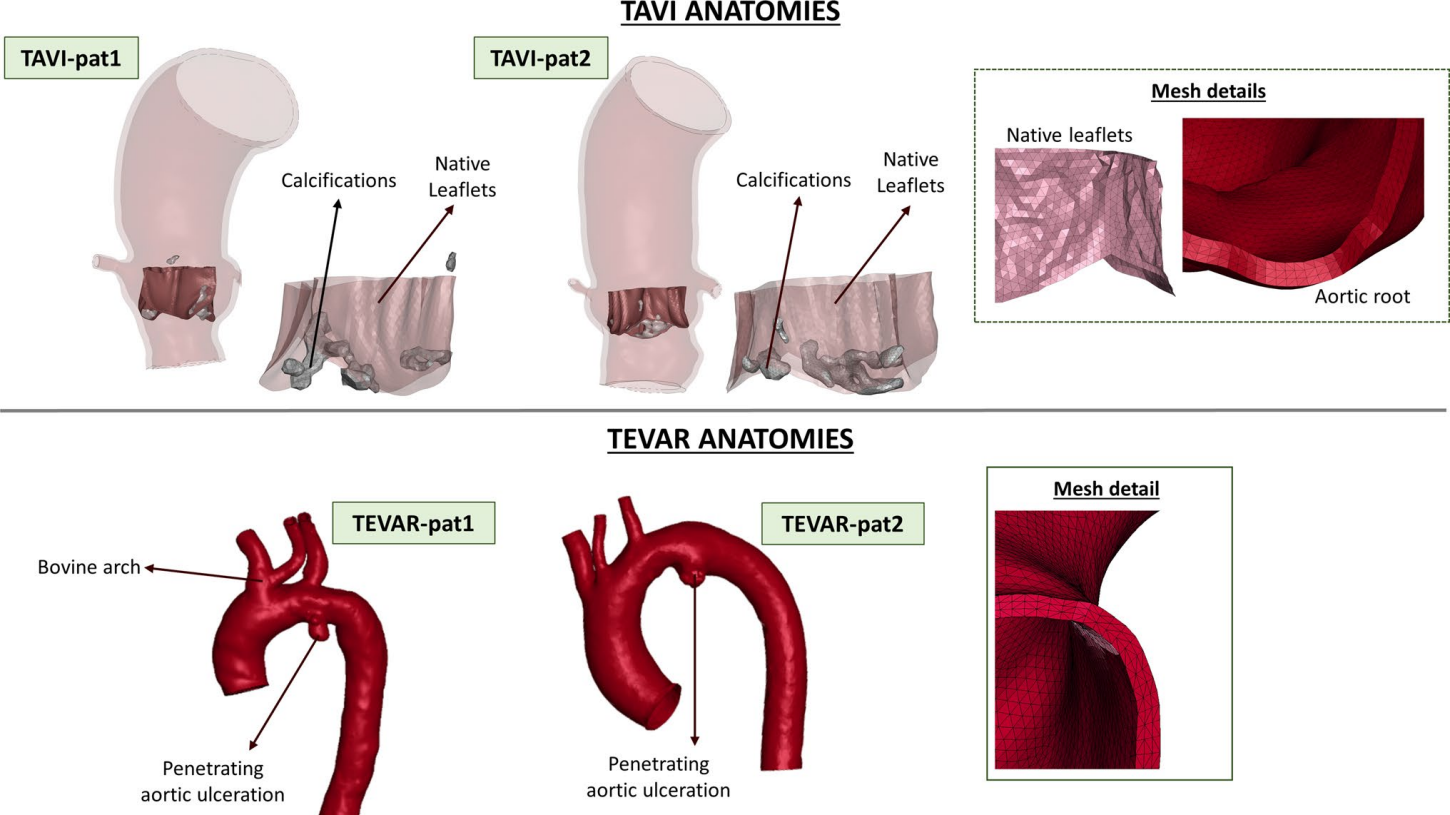
Segmented anatomies and mesh details for TAVI (top) and TEVAR (bottom) simulations

Vessel STL models were obtained through a semi-automatic segmentation of pre-operative CTA images performed with VMTK (Orobix srl, Italy). The aortic roots used in TAVI simulations included the Valsalva sinuses, the origin of the coronary vessels, and the native stenotic aortic valve with calcifications. The aortic anatomies used in TEVAR simulations comprised the aortic arch, the three supraortic vessels, the descending thoracic aorta, and the pathological region. In both cases, solid meshes were obtained using ANSA Preprocessor v23.0.1 (BETA CAE Systems, Switzerland). The same meshing strategy was adopted for all the anatomies: The segmented surface models were discretized with triangular elements and then extruded to create three layers of tetrahedral elements (Fig. 1). Since it was impossible to determine the wall thickness from CTA images, this thickness was assigned the values reported in the literature (Choudhury et al. 2009; Mensel et al. 2014; Hardikar et al. 2020).

Table 1 reports the aortic mesh details in terms of element size, assigned thicknesses, and the total number of tetrahedral elements in the four anatomies. The original valve leaflets were discretized with quadrangular shell elements (element size of 0.45 mm, thickness of 0.1 mm); while, calcifications were discretized with tetrahedral elements (element size of 0.8 mm). Material models and parameters adopted for the different components of the anatomies together with the literature references are listed in Table 2. Comparison tests with a linearized material model for the aortas were also carried out and results are reported in the Supplementary materials).

**Table 1:** Mesh details of the four anatomies included in the study (abbreviations: pat = patient)

Anatomy	Element size	Thickness	# of tetrahedral elements
TEVAR-pat1	0.8–1 mm	1 mm (supraortic arteries) to 1.8 mm (aortic arch and descending aorta)	617,950
TEVAR-pat2			619,920
TAVI-pat1	0.5–0.8 mm	0.7 (coronary vessels) to 2.1 mm (aortic root and aorta)	353,227

**Table 2:** Mechanical properties of the different components of the aortic anatomies and devices with the literature references

	Component	Material model	Material parameters (Mpa)	References
TEVAR	Aorta	Yeoh—2nd order	C10 = 0.077 MPa C20 = 0.836 MPa	Raghavan and Vorp (2000)
		Linearization	*E* = 2 MPa	
	Stent (device)	Super-elastic Shape-memory alloy	*E*_A_ = 57.5 MPa *ν* = 0.3 *E*_M_ = 47.8 MPa *ε* = 0.0063 [ −] *σ*_L_^S^ = 550 MPa *σ*_L_^E^ = 620 MPa *σ*_U_^S^ = 450 MPa *σ*_U_^E^ = 250 MPa	Ramella et al. (2022)
	Graft (device)	Fabric linear elastic	*E* = 1080 MPa *ν* = 0.35	
TAVI	Aortic root	Yeoh—3rd order	C10 = 0.041 MPa C20 = 0.118 MPa C30 = 0.455 MPa	Azadani et al. (2012)
		Linearization	*E* = 1.1 MPa	
	Native leaflets	Yeoh—2nd order	C10 = 0.008 MPa C20 = 0.048 MPa	Stradins et al. (2004)
	Calcifications	Linear elastic	*E* = 12.6 MPa *ν* = 0.45	Holzapfel et al. (2004)
	Stent (device)	Super-elastic Shape-memory alloy	*E*_A_ = 44 MPa *ν* = 0.3 *E*_M_ = 20 MPa *ε* = 0.055 [ −] *σ*_L_^S^ = 460 MPa *σ*_L_^E^ = 465 MPa *σ*_U_^S^ = 360 MPa *σ*_U_^E^ = 260 MPa	Finotello et al. (2021)
	Leaflets (device)	Linear elastic	*E* = 2.15 MPa *ν* = 0.45	Caballero et al. (2017)
	Skirt (device)			

### Pre-stress

The anatomies obtained from the segmentation of gated CTA images, corresponding to the diastolic pressure, represent a stressed configuration of the vessels (denoted CT configuration, CTC). Therefore, an accurate FE analysis requires first to identify a stress-free reference configuration of the vessel. In this work, the stress-free reference configuration is assumed to correspond to the shape of the vessel at 0 mmHg and is denoted as the zero-pressure configuration (ZPC). The inverse elastostatic method (Govindjee and Mihalic 1996) already implemented within the ANSYS Mechanical FEA software (Ansys Inc., Canonsburg, PA, USA), was used to obtain the ZCP of the vessel from the CTC. The methodology consists in using the inverse solver for nonlinear static structural analysis by applying the diastolic pressure—assumed to be 80 mmHg (Kan et al. 2021)—on the internal lumen of the CTC to deflate it obtaining the ZPC configuration. All vessel outlets are assumed to be fixed in space (no translation and rotation allowed). This nonlinear analysis uses the full Newton–Raphson solution procedure, an implicit iterative method. Inverse solving is only supported for static structural nonlinear analysis with the large deflection property on. The automatic time stepping feature was adopted (0.05 s). Material parameters used for the aortic wall modeling are reported in Table 2.

**Fig. 2:**
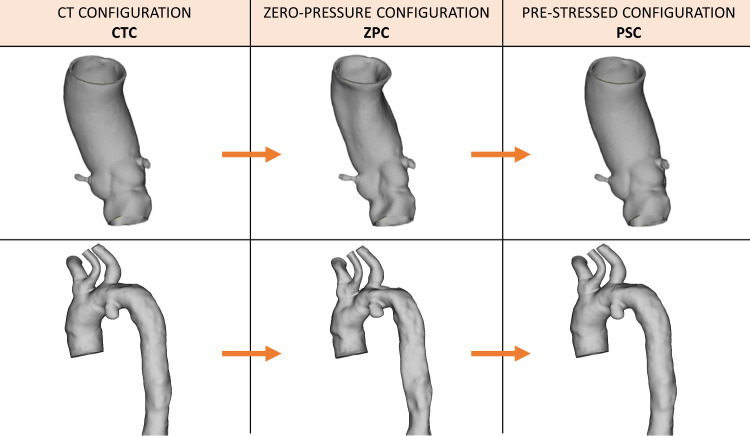
Schematic representation of the pre-stress simulation: from the segmented CT image, CTC, the zero-pressure configuration, ZPC, is obtained and then it is loaded at the diastolic pressure to obtain the pre-stress configuration, PSC. Only TAVI-pat1 and TEVAR-pat1 are depicted, but the same stands for the other two anatomies

The obtained ZPCs were then used as the reference configurations for all subsequent analyses. The stressed configuration obtained when the ZPC is loaded with the diastolic pressure is denoted as the pre-stressed configuration (PSC). A scheme of the methodology is depicted in Fig. 2.

The obtained PSCs were validated by comparing them with the CTC in terms of nodal position. The nodal position percentage error was calculated for each anatomic model included in the study, as follows:

$$\mathrm{RMS\%}=\sqrt{\frac{\sum_{i}{\left({N}_{{\mathrm{PSC}}_{i}}-{N}_{{\mathrm{CTC}}_{i}}\right)}^{2}}{\sum_{i}{\left({N}_{{\mathrm{CTC}}_{i}}\right)}^{2}}}\cdot 100$$

where ${N}_{{\mathrm{PSC}}_{i}}$ represents the spatial position of the nodes in the PSC and ${N}_{{\mathrm{CTC}}_{i}}$ their position in the CTC.

### TAVI and TEVAR simulations

**Fig. 3:**
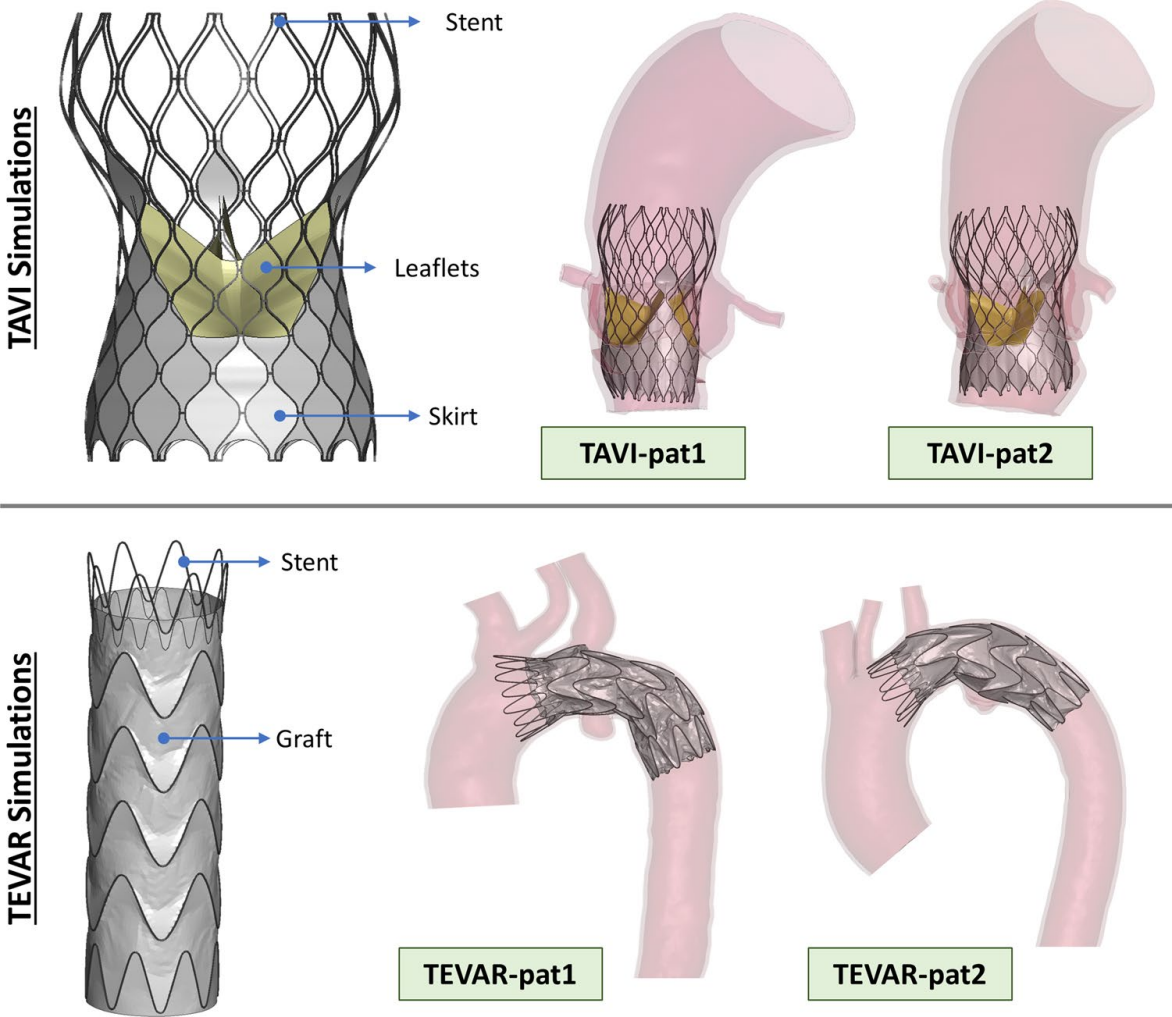
Top: transcatheter valve model and final TAVI simulation configuration for TAVI-pat1 and TAVI-pat2. Bottom: stent-graft model and deployed configuration after TEVAR simulations for TEVAR-pat1 and TEVAR-pat2

The transcatheter valve CoreValve Evolut R was modeled with SolidWorks2018 (Dassault Systèmes SolidWorks Corp., Waltham, MA, USA). It was composed of the stent, the leaflets, and the skirt, as depicted in Fig. 3. The stent was discretized with 5340 Huges-Liu beam elements; the leaflets were discretized with 3840 quadrilateral linear shell elements; whereas, 29,538 triangular membrane elements were used to discretize the thin skirt. Material parameters are reported in Table 2.

TAVI simulations were based on three steps as reported in Luraghi et al. (2019). First, the device was crimped inside a rigid catheter. The device was then placed in an intermediate crimped configuration by releasing it inside a second rigid catheter with a diameter of 10 mm coaxially positioned with the valve annulus. In the last phase, the rigid catheter was unsheathed distally to let the device gradually open against the annulus wall as it happens during the operation. Simulations were performed with the CTC and the PSC for both patients (Fig. 3).

The free-flow Valiant Captivia stent-graft was adopted in the simulation and was modeled following the work by Ramella et al. (2022) (Fig. 3): The stent and the graft were, respectively, discretized with 1232 Huges-Liu beam elements and 16,414 triangular membrane elements. Table 2 reports the adopted material parameters.

TEVAR FE simulations comprised three phases following a recently validated methodology (Ramella et al. 2022, 2023). The device was initially crimped inside a catheter, then displaced up to the proximal landing zone along the vessel centerline, and, finally, it was progressively deployed into the vessel starting from the proximal end. For each aortic anatomy, stent-graft deployment was simulated both accounting and not accounting for pre-stress in the aortic wall. Figure 3 shows the deployed device configuration in the two patients.

Technical details of simulations (i.e., contacts, boundary conditions) are reported in the Supplementary materials. All the TAVI and TEVAR finite element simulations were performed on 28 CPUs of an Intel Xeon64 with 250 GB of RAM using the commercial explicit finite element solver LS-DYNA 971 Release 14.0 (ANSYS, Canonsburg, PA, USA). A selective mass scaling was adopted in order to have a constant time-step of 0.001 ms. The post-processing analysis was carried out with META Postprocessor v23.0 (BETA CAE System, Switzerland).

Simulation results were analyzed in terms of normal contact pressure on the vessel wall, device-to-vessel distance, von Mises stress distribution in the nitinol stent, and von Mises stress and strain in the aorta. The evaluation of normal contact pressure and device-to-vessel distance aimed at assessing the outcome of the simulated surgical procedure, the quality of the device apposition to the aortic wall, and the interactions between the stent and the vessel. These markers served as indicators of the possible device migration which can impact the procedural success. The stent stress state, both for the transcatheter valve and stent-graft, was assessed as an indicator of the risk of nitinol device rupture and fatigue life predictor. Furthermore, aortic stresses and strains originating after the device implantation were evaluated as indicators of possible vascular damage resulting from the procedure (Hemmler et al. 2019a, b; Barati et al. 2022).

## Results

### Aortic wall pre-stress simulation results

**Fig. 4:**
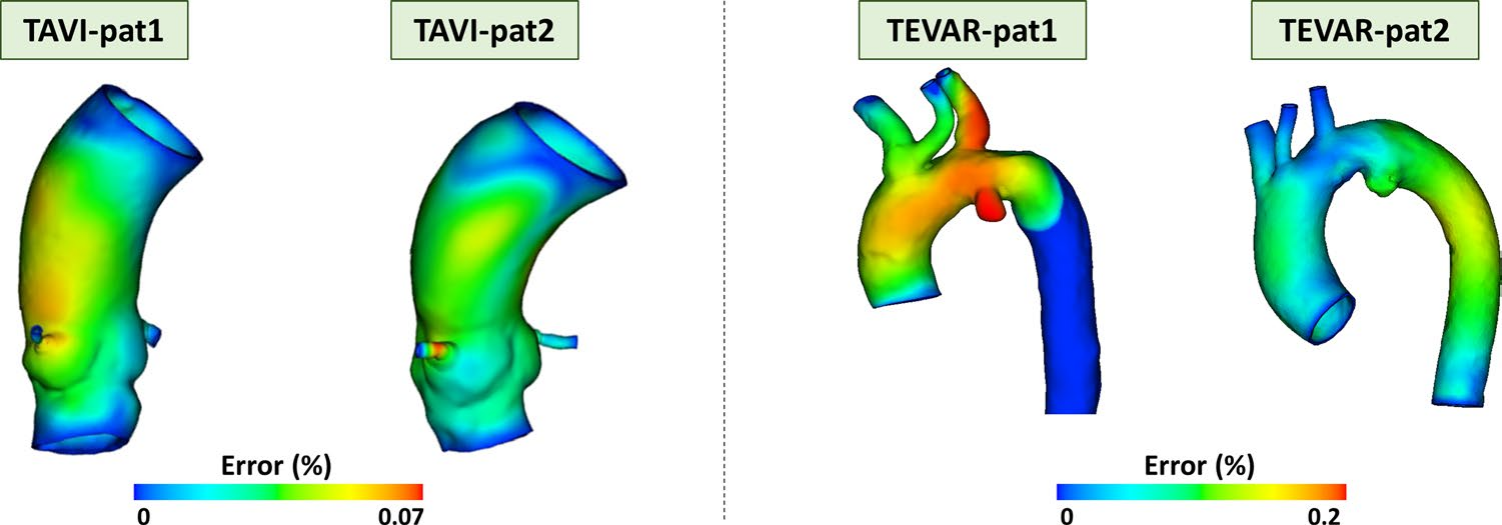
Percentage error distribution between the CT configuration, CTC and pre-stressed configuration, PSC, after re-pressurization from the zero-pressure configuration, ZPC

Figure 4 shows the percentage nodal position error distribution in the investigated anatomies. For TAVI, percentual errors were below 0.07%, while for TEVAR the largest error reached a value of 0.21%. In both cases, higher errors were located on the external curvature of the aorta and at the level of the pathology.

### TAVI simulations

**Fig. 5:**
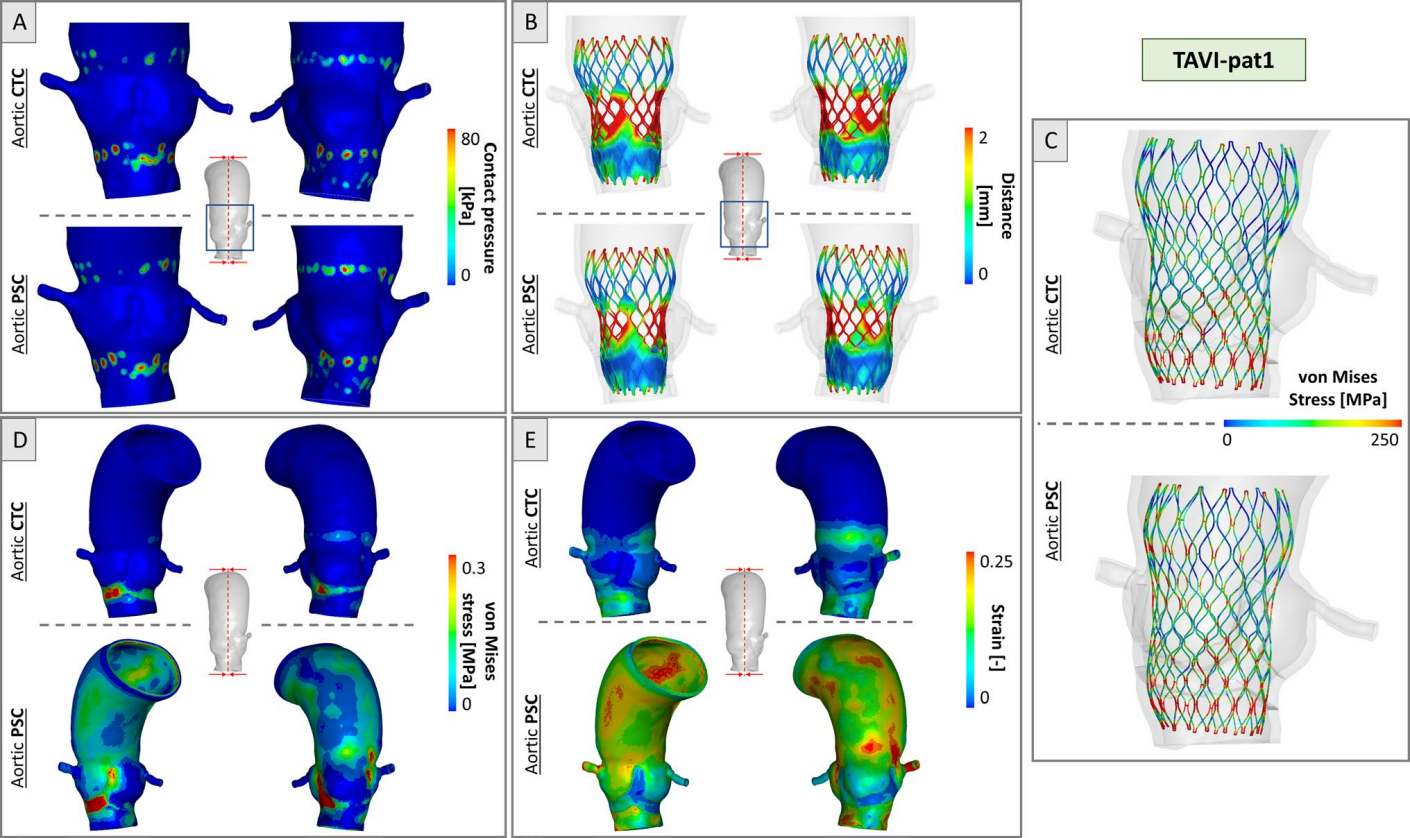
Simulation results for TAVI-pat1 for CTC and PSC cases at the end of deployment. **a** Contact pressure distribution; **b** device-to-aorta distance plotted on the device; **c** von Mises stress distribution in the stent struts; **d** von Mises stress distribution in the aortic wall and **e** strain distribution in the aortic wall

**Fig. 6:**
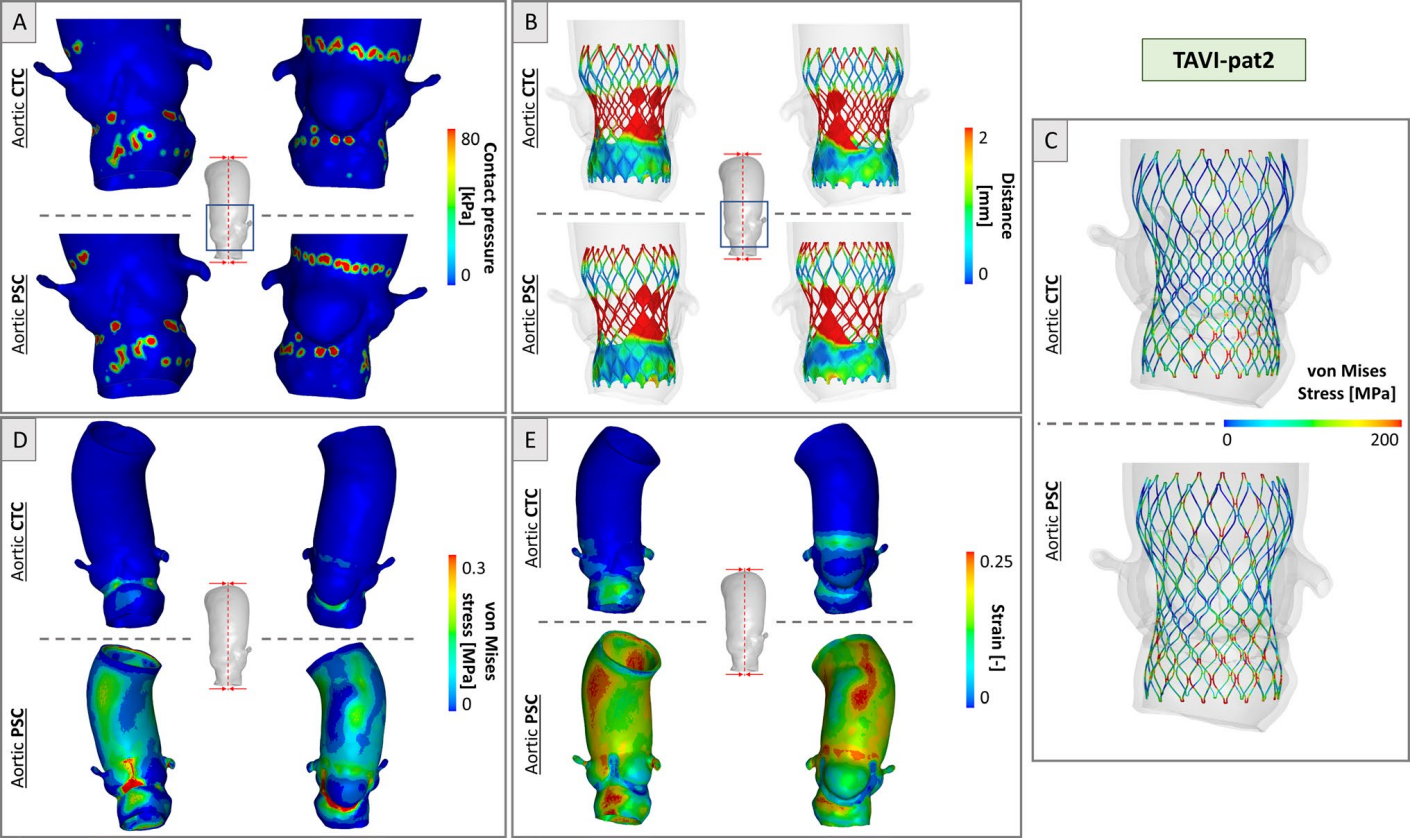
Simulation results for TAVI-pat2 for CTC and PSC cases at the end of deployment. **a** Contact pressure distribution; **b** device-to-aorta distance plotted on the device; **c** von Mises stress distribution in the stent struts; **d** von Mises stress distribution in the aortic wall and **e** strain distribution in the aortic wall

Figures 5 and Fig. 6 report the TAVI simulation results for the two anatomies. Contact pressure distribution was localized in the stent contact zones, with higher values registered in PSC (Figs. 5, 6a). Peak values of 439 kPa and 229 kPa were found in the TAVI-pat1 and TAVI-pat2, respectively, with PSC, versus a value of 344 kPa and 154 kPa in the case of CTC. This latter consideration highlights how the CTC underestimates the contact pressure of about 48% in TAVI-pat2.

Regarding the valve-to-aorta distances, in both anatomies, a lower distance (< 0.3 mm) in the annulus region was achieved, while higher values (> 2 mm) were found at the level of the aortic root where the valve is not in contact with the vessel (Figs. 5, 6b). In the PSC cases, lower mean distances of 3% were detected in the annulus region, with respect to CTC models.

Regarding the stress distribution in the stent, the most stressed zones were localized in the junctions of adjacent cells, with values reaching 213 MPa for TAVI-pat1 and 344 MPa for TAVI-pat2, in the case of CTC vessels. On the contrary, higher and more uniformly distributed stresses with peak values of 231 and 389 MPa were observed in TAVI-pat1 and TAVI-pat2, respectively, in the case of PSC aortas, because the vessels are more rigid due to the inclusion of pre-stress (Figs. 5, 6c).

With CTC vessels, the maximum stress in the tissue was limited to the aortic annulus, and it was due to contacts between the device and the vessel. For both patients, the peak values were positioned in the annulus region. In CTC, stresses reached the maximum values of 1.22 MPa and 0.48 MPa for TAVI-pat1 and TAVI-pat2, respectively. In the case of aortic PSCs, maximum stress values of 2.02 MPa and 1.73 MPa were reached for TAVI-pat1 and TAVI-pat2, respectively. The exclusion of the pre-stress underestimated largely the aortic stress (Figs. 5, 6d).

Regarding the strains, the maximum deformations recorded were 22.9% and 39.7% for TAVI-pat1 and 17.1% and 31.1% for TAVI-pat2 without (CTC) and with aortic pre-stress (PSC) (Figs. 5, 6e).

### TEVAR simulations

**Fig. 7:**
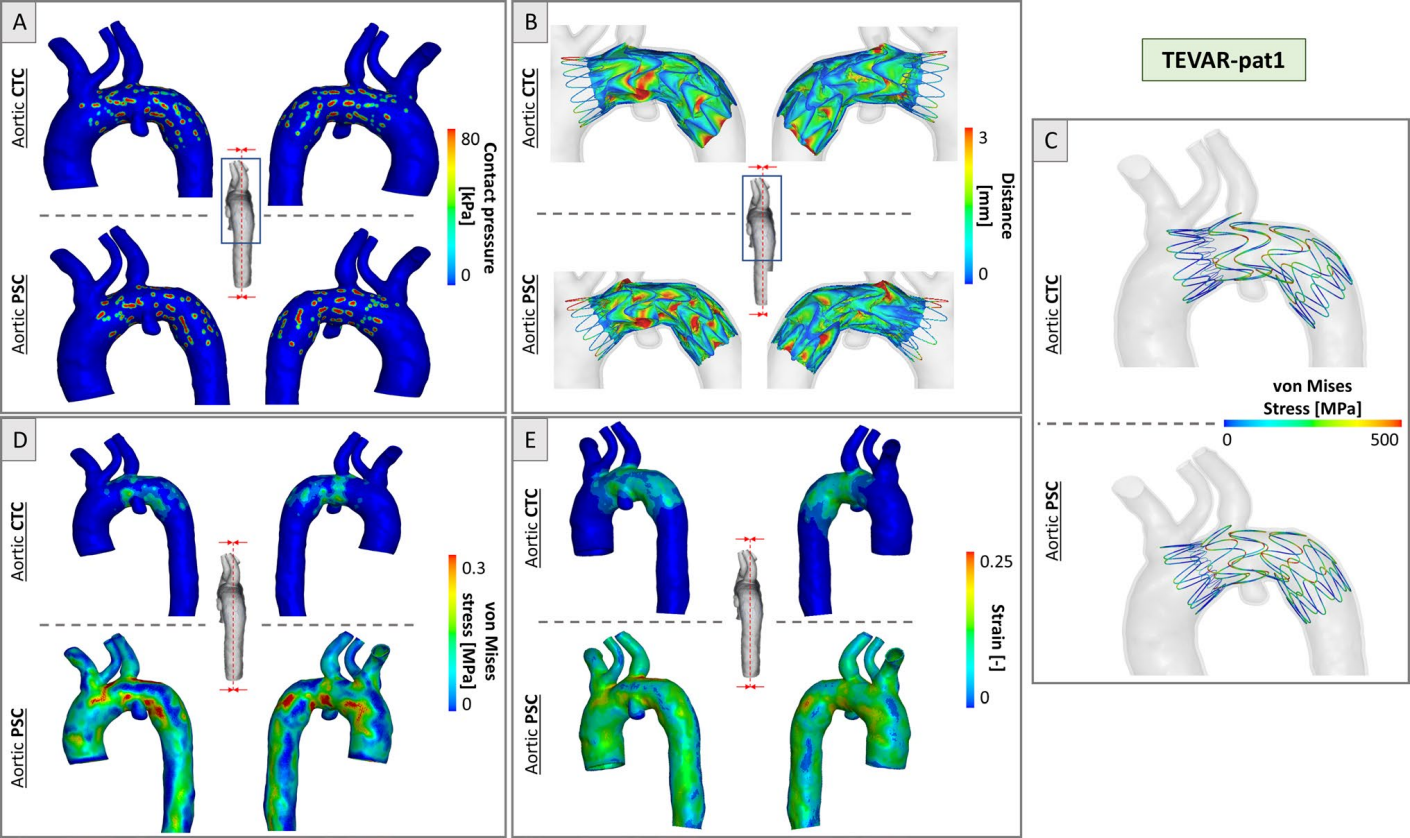
Simulation results for TEVAR-pat1 in CTC and PSC cases at the end of deployment. **a** Contact pressure distribution; **b** device-to-aorta distance plotted on the device; **c** von Mises stress distribution in the stent struts; **d** von Mises stress distribution in the aortic wall and **e** strain distribution in the aortic wall

**Fig. 8:**
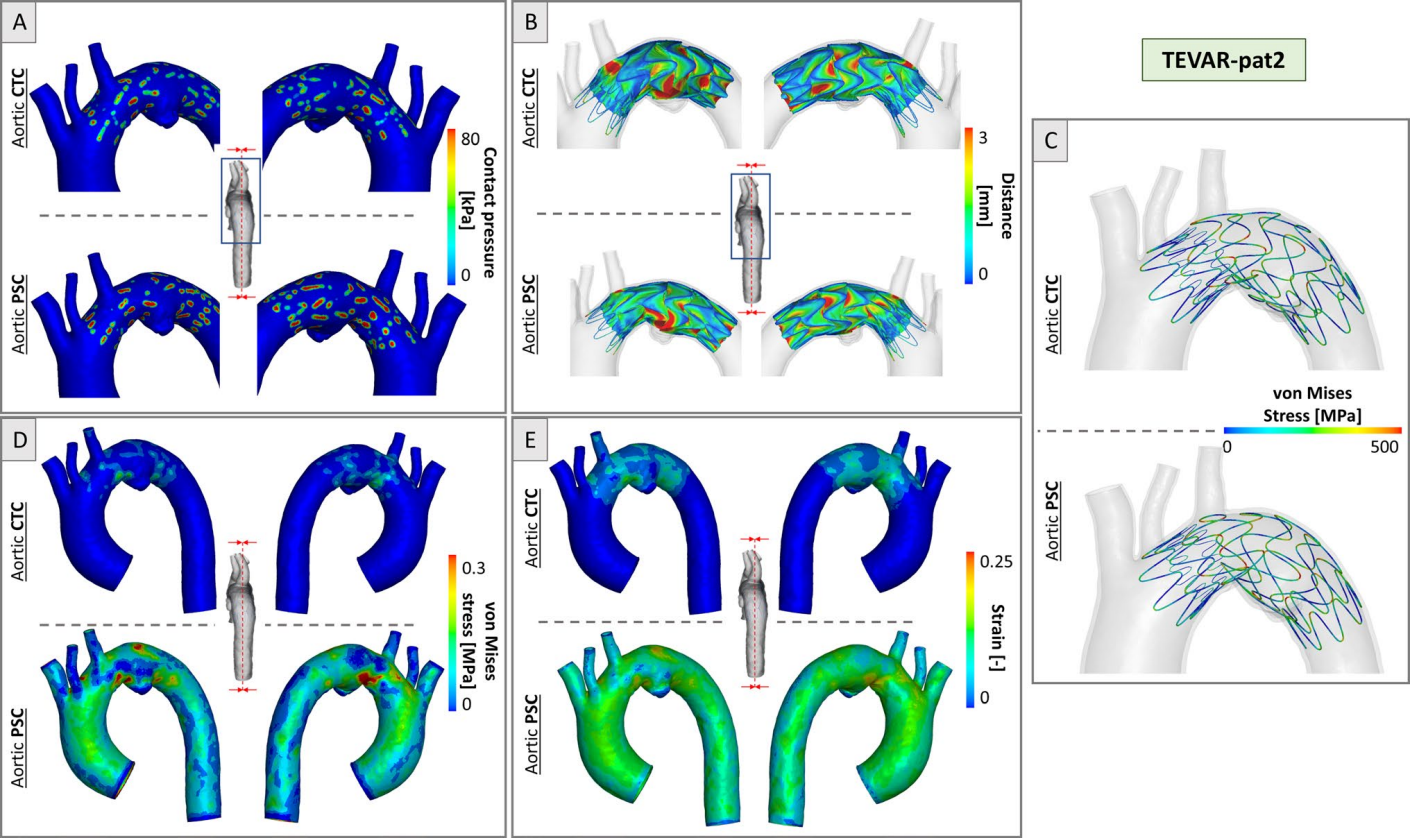
Simulation results for TEVAR-pat2 in CTC and PSC cases at the end of deployment. **a** Contact pressure distribution; **b** device-to-aorta distance plotted on the device; **c** von Mises stress distribution in the stent struts; **d** von Mises stress distribution in the aortic wall and **e** strain distribution in the aortic wall

Figures 7 and 8 depict the TEVAR simulation results. As reported in Figs. 7, 8a, contact pressures reached higher values where the stent struts were in contact with the vessel, in particular at the internal curvature of the vessel. On average, the contact pressure resulted in 55% higher in simulations based on the PSCs of the aorta, i.e., 81 kPa in the CTC versus 125 kPa in the PSC.

Regarding the distance between the stent-graft and the aorta, stent struts showed a good apposition to the vessel with an average distance of less than 3 mm, both in CTCs and PSCs. In both anatomies, distances greater than 3mm were found in the vessel’s narrow curvature regions, in proximity to the supra-aortic vessel root, and in correspondence with the diseased zone. Regarding the graft, the distance between the fabric and the aorta was more inhomogeneously distributed with, on average, larger values with respect to the stent struts due to fabric foldings (Figs. 7, 8b).

Regarding the stress distribution in the stent, a similar von Mises stress distribution was reported in the two anatomies with CTC and PSC scenarios. Peak values of 579 MPa (TEVAR-pat1) and 584 MPa (TEVAR-pat2) were reported when PSC was accounted for versus values of 563 MPa (TEVAR-pat1) and 570 MPa (TEVAR-pat2) in CTC simulations (Figs. 7, 8c).

Regarding the aortic wall, differences between simulations with (PSC) and without (CTC) pre-stress configurations were observed in both patients. By analyzing the von Mises stress distributions at the end of deployment simulation, PSC aortas were subjected to higher stress values, in particular at regions adjacent to the supra-aortic vessels (maximum of 1.23 MPa for TEVAR-pat1 and 0.81 MPa for TEVAR-pat2 at the end of the simulation). Conversely, CTC aortas exhibited non-zero stress values only in the regions in contact with the stent struts, with higher values observed on the internal curvature (maximum of 0.37 MPa for TEVAR-pat1 and 0.44 MPa for TEVAR-pat2). Regarding the strains, the maximum deformations recorded were 22.9% and 27.3% for TEVAR-pat1 and a maximum of 23.7% and 29.2% for TEVAR-pat2, without and with aortic pre-stress, respectively (Figs. 7, 8d–e). As for stress, different strain distributions were achieved in PSC cases.

## Discussion

When performing FE simulations replicating surgical techniques, such as TAVI and TEVAR, the use of patient-specific aortic anatomies enables investigating the effects that the mechanical characteristics of the device and tissue have on the outcome of the surgical procedure. To reliably perform this process, it is important to include in the numerical model also the pre-stress state present in the vessel when the CTA/MR images are acquired. The current study simulates the deployment of TAVI and TEVAR according to clinical procedures using finite element (FE) simulations in patient-specific anatomies, following the framework proposed by Luraghi et al. (2019) and Ramella et al.(2022), respectively. Simulations were conducted in two scenarios, one considering the pre-stress state of the aorta and the other not considering it. The primary objective was to explore how the inclusion of the aortic pre-stress affects the interaction between the aorta and the implanted devices. Through this investigation, we aim at demonstrating whether excluding pre-stress is an acceptable simplification or if it could lead to inaccurate predictions of vessel/device interaction or other complications. This study is the first one that incorporates aortic pre-stress in TAVI simulations. In fact, to the best of the authors’ knowledge, TAVI FE simulations comprising aortic wall pre-stress are not reported in the literature.

In our study, starting from the segmented configuration (CTCs), we obtained zero-pressure configurations (ZPCs) by considering a diastolic pressure of 80 mmHg. Subsequently, re-pressurization simulations were conducted to obtain the pre-stressed configurations (PSCs). To validate the obtained PSC geometries, we compared the PSCs with the segmented configurations (CTCs), achieving global errors of less than 0.2%. Then, we carried out device implantation simulations in both PSC and CTC cases and evaluated various markers that could be associated with potential complications related to TAVI and TEVAR procedures, such as stent rupture or vessel damage (Hemmler et al. 2019b; Barati et al. 2022). Simulation results analysis and corresponding conclusions are summarized in Table 3.

**Table 3:** Influence of the presence of the aortic PSC on the simulation results for the quantities analyzed as indicators of clinical procedures outcomes

Procedure	Distance device/wall	Contact pressure	Stent stress	Aortic stress	Aortic strain
TEVAR	Similar outcome between PSC and CTC	Higher with PSC (+ 55%) than CTC	Similar outcome between PSC and CTC (+2.5% with PSC)	Higher with PSC (+157%) than CTC Different locations of peak values between PSC and CTC	Higher with PSC (+ 21%) than CTC
TAVI	Lower with PSC than CTC, especially in the annulus region	Higher with PSC (+ 48%) than CTC	Higher with PSC (+48%) than CTC	Higher with PSC (+162%) than CTC Same location of peak values between PSC and CTC	Higher with PSC (+ 77%) than CTC

*PSC* pre-stressed configuration, *CTC* CT configuration

To assess the quality of stent-graft/valve apposition to the aortic wall, we analyzed normal contact pressures and distances. Higher contact pressures and lower distances indicate a good device positioning and reduced risk of device migration or complications such as paravalvular leakage (TAVI) and endoleaks (TEVAR) (Wei et al. 2018; Hemmler et al. 2019b). Including the PSC in the model, a lower device-wall distance was observed with the transcatheter valve. While in the case of TEVAR patients, almost no difference in device-to-wall distance between the CTC or PSC setup was observed. Our simulations demonstrated good apposition between the stent-graft/valve and the aortic wall in the CTC and PSC scenarios for all simulated cases. This suggests that neglecting the PSC in the model does not negatively impact the apposition quality for TEVAR, whereas for TAVI a different deployed configuration was predicted when including PSC. Upon analyzing the contact pressure distribution, it was found that neglecting the pre-stress in the aorta resulted in an underestimation of the contact pressure of up to 48% for TAVI and 55% for TEVAR applications. This is because the material nonlinearities lead to a larger tangent elastic modulus in the PSC model with respect to the CTC model. Note that in the case of a linearly elastic material, these differences will not be observed.

The von Mises stress state of the nitinol stent (both for valve and stent-graft) was assessed to indicate the risk of device rupture. The stress values observed in the devices were below the limits of rupture or plasticity (Barati et al. 2022) in both TAVI and TEVAR applications. However, there were notable differences in stress distribution between the two devices. Higher stress levels in the valve stent were obtained in the PSC simulations, with an average increase of 48% compared to the CTC simulations. On the contrary, in the stent-graft struts, similar stress values and distributions were observed for the PSC and CTC cases, with an average increase of only 2.5% in the PSC simulations. The differences in stress values between the two devices can be attributed to the aortic annulus being more sensitive to the stiffening caused by the pre-stress, which might have limited the expansion of the device.

Another important result was related to the aortic stress state before and after the implantation. Even if the results may seem obvious because in the CTC no diastolic pressure is considered, we analyzed them because several studies in the literature speculate on stress distributions from a stress-free vessel domain. As expected, PSC reported higher aortic wall stresses with respect to CTC both before and after device implantation. On average, after the expansion of the device, PSC cases exhibited a 162% increase in aortic wall von Mises stresses for TAVI and a 157% increase for TEVAR compared to the CTC one. For TAVI anatomies, the peak value of the stress was located in the same regions both in the CTC and PSC aortas. These regions were specifically the areas in contact with the stent. However, in the PSC scenario, the stress values were higher and more uniformly distributed throughout the lumen of the vessel. Instead, this is not true for TEVAR simulations. While in CTC, non-zero stress values were found only in the regions in contact with the stent ring, in PSC anatomies, stress peak values were located near the supra-aortic vessels root or on the internal curvature of the aortic arch. The difference in stress distribution between the two procedures can be attributed to the peculiarities of the aortic geometry used for TEVAR simulations, namely the presence of supra-aortic branches: They influenced the pre-stress patterns. They resulted in stress concentrations in diverse locations compared to the CTC scenario. This indicates that the aortic anatomy primarily affects stress distributions within the aorta and highlights the importance of considering individual anatomical characteristics. Focusing on the strain distribution in TAVI applications, in both patients of our study, it was possible to observe a higher maximum deformation when pre-stress is accounted for. In particular, aortic root strains reached a maximum value of 17.1% with CTC and 31.1% with PC for TAVI-pat2: accounting or not for the pre-stress may lead to a wrong estimation of the atrioventricular block as suggested by Bosi et al. (2020).

The presented work is not exempt from limitations. The vascular anatomies were modeled as isotropic tissues using the literature parameters. This approach assumes uniform material properties throughout the vessel walls and may not fully capture the complexities and variations in tissue behavior in real anatomies. In addition, the wall thickness values were obtained from the literature since it was not possible to extract them from CTA images. Unfortunately, post-op CT images were not available to validate the final configuration of the devices. However, we demonstrated that pre-stress does not impact the TEVAR deployment and does slightly on TAVI deployment. The most important differences in terms of stress and strain cannot be validated by means of non-invasive methods. Pre-stretch was also neglected in this study. However, as suggested by Desyatova et al. (2020), it has no effect on elder patients as the ones considered in this study. Also, one commercial stent-graft and one commercial transcatheter valve were studied: The study can be in future extended to other commercial devices to verify the findings of this work. However, it is worth noting that the study was a proof-of-concept investigation aiming to assess and quantify the influence of aortic pre-stress in TEVAR and TAVI simulations. Finally, a mean value of 80 mmHg was used for all the patients as end-diastolic pressure as patient-specific data were not available. Given the specific focus and goals of the study, these simplifications may be considered acceptable within the scope of the research. Nonetheless, it is essential to consider these limitations when interpreting the results and their applicability to real clinical situations.

In conclusion, the analysis reveals that aortic wall pre-stress minimally impacts the deployed configuration (in terms of device/aorta distance) and stress distribution of the stent-graft and valve. However, it plays a crucial role in accurately capturing the contact pressure and the stress/strain distributions on the aortic wall. By incorporating aortic wall pre-stress, this study enhances the understanding of the biomechanical behavior and interactions between the implanted devices and the aortic wall. It emphasizes the importance of considering pre-stress for more reliable assessments of device-related complications.

### Supplementary Information

Below is the link to the electronic supplementary material.

Supplementary file1 (DOCX 437 kb)
