# Supplementary material for: On the necessity to include arterial pre-stress in patient-specific simulations of minimally invasive procedures
Source: Biomech Model Mechanobiol. 2023 Dec 8;23(2):525–37. doi: 10.1007/s10237-023-01789-0 (PMC10963513; doi:10.1007/s10237-023-01789-0)
Supplement: Supplementary file 1 — Supplementary file1 (DOCX 437 kb) [file 10237_2023_1789_MOESM1_ESM.docx]

Supplementary material

Linearization of the hyperelastic material

For the linearization of the material properties, we hypothesized a linear elastic, isotropic and uncompressible ($\nu=0.49)$ material. Through a FEA analysis, we applied a pressure load corresponding to the physiological blood pressure curve to the internal lumen of the walls characterized with hyperelastic material properties. Fifteen elements were chosen along the vessel: for each of them the first, second and third principal stress ($\sigma_{1}, \sigma_{2}, \sigma_{3}$) and strain ($\varepsilon_{1}, \varepsilon_{2}, \varepsilon_{3})$ were extracted in the diastolic and in the systolic peak and the difference of these values between systole and diastole was computed ($\Delta\sigma and \Delta\varepsilon$). For each element the material properties were linearized as follows:

$$\Delta\varepsilon_{1}=\frac{{\Delta\sigma}_{1}}{E}-\frac{\nu}{E} ( {\Delta\sigma}_{2}+ {\Delta\sigma}_{3})$$

$$\Delta\varepsilon_{2}=\frac{{\Delta\sigma}_{2}}{E}-\frac{\nu}{E} ( {\Delta\sigma}_{1}+ {\Delta\sigma}_{3})$$

$$\Delta\varepsilon_{3}=\frac{{\Delta\sigma}_{3}}{E}-\frac{\nu}{E} ( {\Delta\sigma}_{1}+ {\Delta\sigma}_{2})$$

$$\left[ \begin{matrix} \Delta\varepsilon_{1} \\ \Delta\varepsilon_{2} \\ \Delta\varepsilon_{3} \end{matrix} \right] E= \left[ \begin{matrix} {\Delta\sigma}_{1}-\nu( {\Delta\sigma}_{2}+ {\Delta\sigma}_{3}) \\ {\Delta\sigma}_{1}-\nu( {\Delta\sigma}_{2}+ {\Delta\sigma}_{3}) \\ {\Delta\sigma}_{1}-\nu( {\Delta\sigma}_{2}+ {\Delta\sigma}_{3}) \end{matrix} \right]$$

By solving this system, the elastic modulus for each selected node is calculated as:

$$E= \frac{1}{\Sigma{\Delta\varepsilon_{i}}^{2}} \left\{ \Delta\varepsilon_{1} \left[ {\Delta\sigma}_{1}- \nu( {\Delta\sigma}_{2}+ {\Delta\sigma}_{3}) \right]+ \Delta\varepsilon_{2} \left[ {\Delta\sigma}_{2}- \nu( {\Delta\sigma}_{1}+ {\Delta\sigma}_{3}) \right]+ \Delta\varepsilon_{3} \left[ {\Delta\sigma}_{3}- \nu( {\Delta\sigma}_{1}+ {\Delta\sigma}_{2}) \right] \right\}$$

The final elastic modulus was obtained by averaging the value found for each element. In particular of 2MPa in TEVAR application and 1.1 MPa in TAVI application. Results of the CTC simulations with linearized material are reported in the following table.

| Variable | TEVAR | | TAVI | |
| --- | --- | --- | --- | --- |
|  | Patient 1 | Patient 2 | Patient 1 | Patient 2 |
| Contact pressure [MPa] | 6.0 | 5.2 | 756 | 792 |
| Stent – von Mises stress [MPa] | 592 | 482 | 308 | 385 |
| Aorta – von Mises stress [MPa] | 0.40 | 0.39 | 0.31 | 0.28 |
| Aorta – strain [-] | 0.19 | 0.14 | 0.28 | 0.23 |

TEVAR simulation

*Boundary conditions*

Aortic outlets (ascending aorta, three supra-aortic branches and descending aorta) fixed in the three directions (both displacements and rotations).

*Contacts*

Soft penalty-based contacts were defined between the stent and aorta and between the graft and aorta with a friction coefficient of 0.1 (defined after a sensitivity analysis, as reported in our previous paper [1],[2]). A node-to-node connection was defined between the graft and stent to replicate the presence of the suture points.

TAVI simulation

*Boundary conditions*

Aortic outlets (LVOT, coronary arteries, aortic arch) fixed in the three directions (both displacements and rotations).

*Contacts*

Soft penalty-based contacts were defined as follows based on our previous work [3][4]:

- Stent – aorta (friction 0.2);
- Stent – native valve (friction 0.2);
- Stent – calcifications (no friction);
- Skirt – aorta (no friction);
- Skirt – native valve (no friction);
- Skirt – calcifications (no friction).

A node-to-node connection was defined between the skirt and stent as the two components were attached in the real device.
